# Utilizing IoMT-Based Smart Gloves for Continuous Vital Sign Monitoring to Safeguard Athlete Health and Optimize Training Protocols

**DOI:** 10.3390/s24206500

**Published:** 2024-10-10

**Authors:** Mustafa Hikmet Bilgehan Ucar, Arsene Adjevi, Faruk Aktaş, Serdar Solak

**Affiliations:** 1 Information Systems Engineering, Kocaeli University, Umuttepe Campus, 41001 Kocaeli, Turkey; mhbucar@kocaeli.edu.tr (M.H.B.U.); 235172001@kocaeli.edu.tr (A.A.); 2 Biomedical Engineering, Kocaeli University, Umuttepe Campus, 41001 Kocaeli, Turkey; faruk.aktas@kocaeli.edu.tr

**Keywords:** athlete well-being, vital sign monitoring system, IoMT, physiological parameters, smart glove, training optimization

## Abstract

This paper presents the development of a vital sign monitoring system designed specifically for professional athletes, with a focus on runners. The system aims to enhance athletic performance and mitigate health risks associated with intense training regimens. It comprises a wearable glove that monitors key physiological parameters such as heart rate, blood oxygen saturation (SpO2), body temperature, and gyroscope data used to calculate linear speed, among other relevant metrics. Additionally, environmental variables, including ambient temperature, are tracked. To ensure accuracy, the system incorporates an onboard filtering algorithm to minimize false positives, allowing for timely intervention during instances of physiological abnormalities. The study demonstrates the system’s potential to optimize performance and protect athlete well-being by facilitating real-time adjustments to training intensity and duration. The experimental results show that the system adheres to the classical “220-age” formula for calculating maximum heart rate, responds promptly to predefined thresholds, and outperforms a moving average filter in noise reduction, with the Gaussian filter delivering superior performance.

## 1. Introduction

The real-time monitoring of human physiological functions has gained considerable momentum, particularly in the fields of sports and healthcare. This increasing focus highlights the valuable insights that can be obtained from analyzing the body’s responses during various activities, thereby enabling more effective optimization of training programs and healthcare interventions [1]. The ability to monitor physiological parameters provides a crucial opportunity to enhance athletic performance and overall well-being, making it an essential element in both sports science and health monitoring systems [2,3,4].

**Figure 1:**
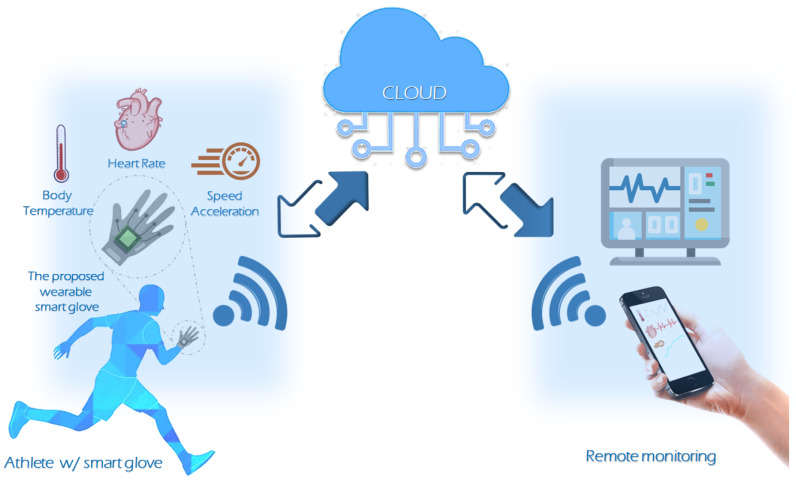
Sports devices and wearables with integrated sensors.

This advancement has been driven by the rapid progress of the Internet of Things (IoT), supported by innovations in sensor technology, communication systems, and personal computing devices. These developments have paved the way for new possibilities in wearable healthcare devices, making sensors more accessible and cost-effective while enabling the real-time monitoring of critical physiological parameters such as functional movements, biomechanical factors, and vital signs [5,6]. For athletes, such data are essential for optimizing performance and reducing injury risk by allowing training plans to be customized to individual needs. Wearable monitoring systems facilitate continuous data collection, enabling the design of precise, personalized training and treatment strategies aimed at injury prevention [7]. Heart rate (HR) is a fundamental physiological metric, extensively utilized to evaluate cardiovascular responses both during physical exertion and throughout recovery phases. Since HRmax is a crucial measure for prescribing exercise intensity and has a strong correlation with maximum oxygen uptake [7], establishing a threshold based on the “220-age” formula [8] is essential for ensuring safe running practices. This threshold helps in providing timely alerts to athletes if their heart rate exceeds the recommended limits, thereby preventing potential health risks. Illustrated in Figure 1 are the applications of IoT in sports, showing the integration of smart sports devices and wearables equipped with embedded sensors.

In our study, the reasons for the use of wearable gloves in athletes, especially runners, are briefly as follows:

Sensor placement: The proposed wearable gloves can offer more precise or stable positioning for certain sensors (e.g., gyroscope) that require better alignment with hand movements for accuracy. For example, while the gyroscope placed on the glove measures speed and acceleration, the sensor placed on the fingertip of the glove measures pulse and SpO2.
Targeted application: The glove can provide better integration of multiple sensors in a single wearable device that captures runner-specific hand movements, which may not be as effective in current wristband-like products on the market. In our study, it is possible to place sensors in appropriate locations on the glove to collect data with high accuracy.
Enhanced measurement: The glove can also allow for more accurate or consistent measurements of hand movement, temperature, or other parameters that are specifically relevant to runners.

In addition to the potential advantages mentioned above, we propose an alternative wearable system for athlete tracking in our study. Moreover, when compared to similar products on the market, the proposed wearable glove approach stands out with its low cost, easy accessibility, and scalability features. The primary objective of this paper is to introduce a wearable glove system equipped with an alarm feature that monitors runners’ speed, heart rate, SpO2, body temperature, and ambient temperature across three distinct phases: resting, walking, and running. The system provides insights into the data collected and alert state based on the monitored parameters during these different phases.

The structure of the paper is as follows: Section 2 provides a review of the existing literature, Section 3 details the proposed system, and Section 4 covers the experimental tests, results, challenges encountered, and future research directions. Finally, Section 5 concludes the paper with a summary of the findings.

## 2. Literature Review

Monitoring athletes’ vital signs during running is essential for optimizing performance and ensuring well-being, providing timely guidance when potential issues arise. Various methods have been developed to track physiological parameters under diverse and challenging conditions. One prevalent approach is the use of wearable smartwatches, which monitor heart rate, SpO2, and hand movement [9,10,11]. Additionally, more specialized systems have been designed, including backpacks equipped with satellite antennas and microcontrollers connected to heart rate sensors worn on the athlete’s finger [12], and similar without satellite communication features, to monitor a variety of physiological parameters through integrated wearable sensors, including heart rate, oxygen saturation, body temperature, blood pressure, and other relevant metrics [13]. Other innovations include smart gloves designed for monitoring fitness exercises [14,15] and combat sports, such as boxing, to aid in mastering defense and punching techniques [16]. Furthermore, body sensor network systems have been developed for mountaineers to enhance safety and performance [17].

Zhao et al. pioneered a wearable sports posture measurement system integrated with Internet of Things (IoT) technology [6]. Through experiments on common sports postures, their system provides critical insights into performance. However, they emphasize the need for caution when assessing heart rate variability (HRV) using non-ECG devices, recommending ECG signals and a minimum respiratory rate of 10 breaths per minute for more accurate HRV analysis in athletes. Similarly Neupert et al. and Karlsson et al. have conducted parallel studies examining athlete monitoring techniques in elite sports, focusing on the United Kingdom [18], as well as acclimatization and training responses of elite cross-country skiers and biathletes at an altitude of 1800 m over a 17–21-day period [19]. Thornton et al. focused their research on the analysis and visualization of athlete data to optimize performance and training strategies [20].

Pawlik et al. conducted practical research on fatigue and training load factors in volleyball, aiming to evaluate both internal and external loads experienced by players during a competitive season [21]. Utilizing accelerometers and subjective assessments like the perceived exertion (RPE) and total quality recovery scale (TQR) questionnaires, they monitored eleven female athletes across five training days. Their results showed that tracking acceleration counts could provide valuable insights into the specific demands placed on volleyball players, aiding in the optimization of training and recovery. Similarly, Rebelo et al. explored this topic with male volleyball athletes, reinforcing the importance of load monitoring in sports [22].

Additionally, innovations such as the system developed by Oktavius et al., which monitors cyclists’ body fatigue by analyzing cortisol levels through a sweat sensor, enable real-time assessments of stress and endurance during cycling performance [23]. Elshafei and Shihab took a different approach by focusing on muscle fatigue monitoring using sensors during repetitive muscle contractions, which is particularly useful for gym and bodybuilding activities [24].

In terms of heart rate monitoring, Huifeng et al. introduced a heart rate sensor designed to track electrical activity in the heart, offering valuable cardiovascular data across various sports disciplines [2]. Zhao and Li expanded this field with the development of a body sensor network (BSN) aimed at monitoring body motion and physiological parameters, which enhances both performance and safety across multiple sports [25].

The use of electromyography (EMG) sensors to track muscle activity has also gained prominence, with Taborri et al. demonstrating how EMG can improve training regimens and prevent injuries by analyzing muscle dynamics during body movements [26]. Meanwhile, Scataglini et al. developed a temperature monitor to assess skin convective heat flux, particularly beneficial for runners in managing heat exposure and optimizing cooling strategies during races [27].

Finally, Gao et al. introduced a cutting-edge wearable sweat analysis technology that monitors not only psychological and physical conditions but also metabolism and body adaptability. This technology has broad applications across different sports and provides comprehensive data to better manage athletic performance [28]. In addition to sports applications, notable advancements have been made in the healthcare sector to address hand-arm vibration syndrome. Aizuddin and Jalil provided a thorough review of this field, highlighting the critical enabling technologies such as sensor and wireless communication technologies. They also proposed a conceptual model for a smart wearable system designed to measure hand-transmitted vibrations, underscoring the potential of such systems in improving healthcare outcomes [29].

In our comprehensive literature survey, it is noteworthy that none of the methods investigated thus far incorporate a built-in alert system. The absence of such a critical feature poses a significant limitation, particularly in the context of athlete monitoring. Real-time alerts play a pivotal role in promptly notifying athletes when potential issues or anomalies arise during training or performance. The integration of a robust alert system is imperative for enhancing the overall effectiveness of athlete monitoring systems, ensuring timely interventions, and ultimately contributing to the optimization of athletic performance and well-being. In light of this gap in existing methodologies, our research endeavors to address this critical need by incorporating an advanced built-in alert system within our wearable technology.

## 3. Proposed System

This section outlines the components of the proposed system, which integrates advanced sensor technologies to provide comprehensive vital sign monitoring for professional athletes. The system features the MPU6050 for motion tracking and the MAX30100 for real-time heart rate and SpO2 measurements, along with other sensors described below. These sensors are seamlessly integrated into a unified framework, enabling efficient data acquisition and transmission.

Additionally, an algorithm has been implemented to analyze the collected data and generate alerts when predefined thresholds for physiological parameters are exceeded. This ensures that any critical changes in the athlete’s health are promptly addressed.

**Figure 2:**
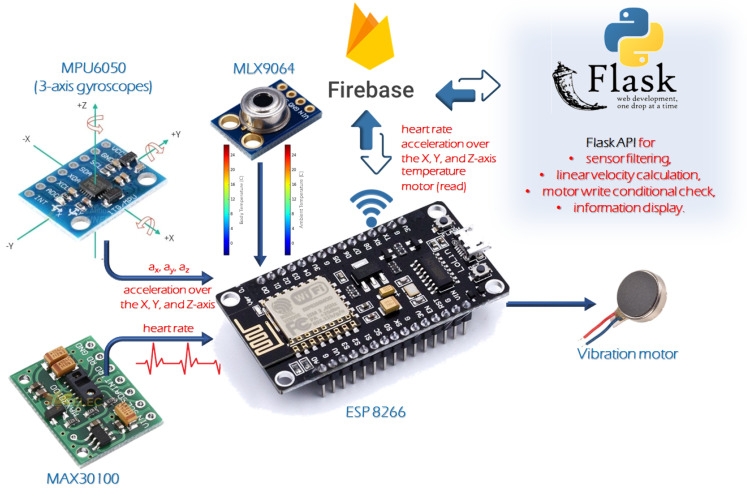
The proposed IoMT-empowered athlete health monitoring and alert system.

Figure 2 presents the block diagram of the proposed system, which incorporates both hardware and software components. The hardware elements include the MAX30100 for the real-time measurement of the pulse, the MPU6050 for the tracking of motion, the vibration motor for the provision of tactile feedback, the MLX9064 for ambient and body temperature measurement, and the ESP8266 for the transmission of data via wireless communication. These hardware components function in conjunction with one another to enable the acquisition, processing, and transmission of data. In addition to the aforementioned hardware components, software elements such as the Firebase database serve to provide the necessary backend infrastructure for the storage and management of the collected data. This comprehensive representation offers a detailed overview of the system’s composition, highlighting the integration of diverse elements to facilitate vital sign monitoring for professional athletes. The entire device is powered via USB cable connected to a PC, enabling both power supply and real-time data transfer through the UART protocol for immediate screening and testing.

### 3.1. Hardware Setup

**Figure 3:**
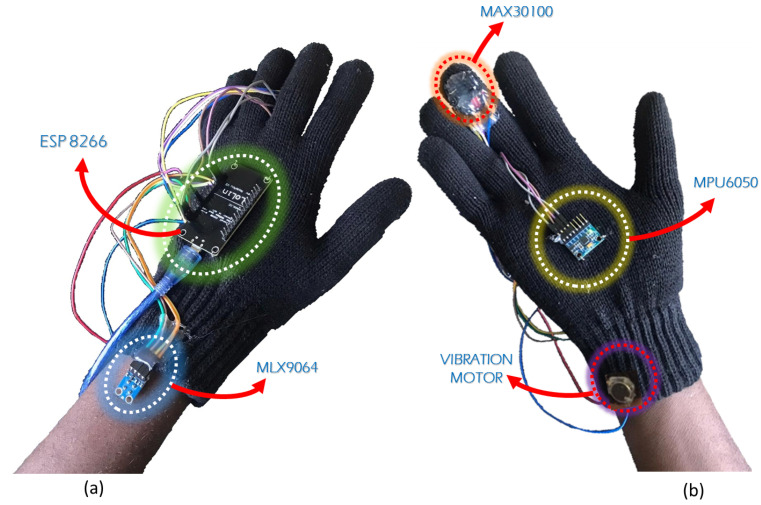
The front (**a**) and back (**b**) views of the prototype IoMT-based athlete health monitoring and alert system.

The front and back views of the proposed smart glove system are shown in Figure 3. The hardware configuration is meticulously designed to capture comprehensive data essential for monitoring an athlete’s physiological parameters during exercise. This integrated hardware setup ensures a robust foundation for our monitoring system, combining precision in data acquisition with real-time communication mechanisms for a comprehensive understanding of an athlete’s health and performance.

As seen in Figure 3, the key components of the hardware setup of the proposed smart glove system consist of ESP8266 as a microcontroller, MAX30100, MPU6050, MLX9064 sensors connected to this microcontroller, and a vibration motor for alert. In this work, the ESP8266 microcontroller is a versatile and powerful component programmed with the Arduino IDE. This microcontroller plays a pivotal role in collecting data from various sensors, including the MPU6050 and pulse sensor. It facilitates the seamless integration of different data streams, ensuring efficient communication between the sensors and the central processing unit. The ESP8266 is also responsible for retrieving real-time heart rate, pulse data, and accurate datetime from an Network Time Protocol (NTP) server. The integration of the MAX30100 into the system adds a crucial dimension to the hardware setup. This component continuously provides real-time heart rate and SpO2 data, a vital metric for evaluating an athlete’s physiological response during exercise. Its seamless incorporation ensures accurate and timely monitoring of the cardiovascular aspect of the athlete’s performance. The MPU6050 sensor is employed to monitor the athlete’s movement by capturing accelerometer and gyroscope data on the x, y, and z axes. This sensor offers invaluable insights into the dynamic aspects of physical activities, enabling a comprehensive analysis of the athlete’s motion and posture during different exercises. The MLX9064 sensor is utilized for measuring body and ambient temperatures, providing critical environmental data for assessing the athlete’s thermal status during activity. The vibration motor integrated into the system functions as an alert mechanism that increases the interaction of the hardware. It provides immediate feedback to the athlete by vibrating when anomalies such as a high heart rate or velocity exceeding the threshold are detected.

### 3.2. Data Collection and Transmission

In this work, the ESP8266 microcontroller plays a pivotal role in the seamless collection and transmission of data. Upon gathering relevant information, the ESP8266 meticulously processes the collected data, employing an efficient algorithm tailored to the specific requirements of the application. This algorithm ensures precision and accuracy in the treatment of data, laying the foundation for robust and reliable subsequent analyses.

The transmission of processed data is facilitated through a secure connection to the Firebase Real-time Database (RTDB), a cloud-based platform known for its scalability and real-time synchronization capabilities. This integration allows for the swift and efficient transfer of data to the cloud, creating a centralized repository for storage and retrieval. The Firebase RTDB serves as a dynamic and responsive backend, accommodating the influx of data in real-time, which is crucial for applications demanding instantaneous updates and monitoring.

To ensure the temporal precision of our data collection, our system integrates an NTP server. Given that ESP8266 lacks a real-time clock module, we rely on retrieving date and time information from an online NTP server when the device is connected to Wi-Fi. This integration plays a pivotal role in furnishing accurate timestamping and synchronization, ensuring coherence among data points.

For the device to interact with the Firebase RTDB, several specific steps are necessary. On the device side, we first ensure the latest library version is installed on the Arduino, enabling communication with the Firebase endpoint for data transmission and reception. To facilitate dynamic Wi-Fi connection, we utilize the Wi-Fi manager library, allowing us to input Wi-Fi credentials at runtime rather than hardcoding them, thus enabling connectivity to any available Wi-Fi access point. Once successfully connected to Wi-Fi and after verifying sensor connectivity, data transmission commences at one-minute intervals. This frequent interval is crucial for promptly detecting any fluctuations in the athlete’s vital sign status.

On the Firebase side, as per the library documentation, data storage at a designated node in the Firebase RTDB involves using specific functions such as set, setInt, setFloat, setDouble, setString, setJSON, setArray, setBlob, and setFile. In our case, we utilize setFloat for parameters like acceleration (x, y, z), gyroscope readings (x, y, z), temperatures, heart rate (BPM—beats per minute), SpO2, and timestamps (in UTC format), while setInt is employed for alert status. Retrieval of these data points is facilitated using the getFloat and getInt functions, respectively.

Authentication credentials are imperative for accessing our Firebase RTDB. Hence, we establish a username–password combination, alongside utilizing the Firebase API key in the connection handler on the device side. Furthermore, we implement a design modification by modifying the rules to restrict access solely to authenticated users, ensuring data security and integrity. The user can only access the database nodes under the node with its user UID. Each node within the database holds pertinent data accessible through designated paths such as sensor/acc/x, sensor/acc/y, sensor/acc/z, sensor/gyro/x, sensor/gyro/y, sensor/gyro/z, sensor/temp, sensor/BPM, and timestamp, alongside/sensor/alert. The ’alert’ node facilitates binary control, accepting values of 1 or 0 to initiate or halt alerts the vibration motor.

### 3.3. Web Interface and Backend API

**Figure 4:**
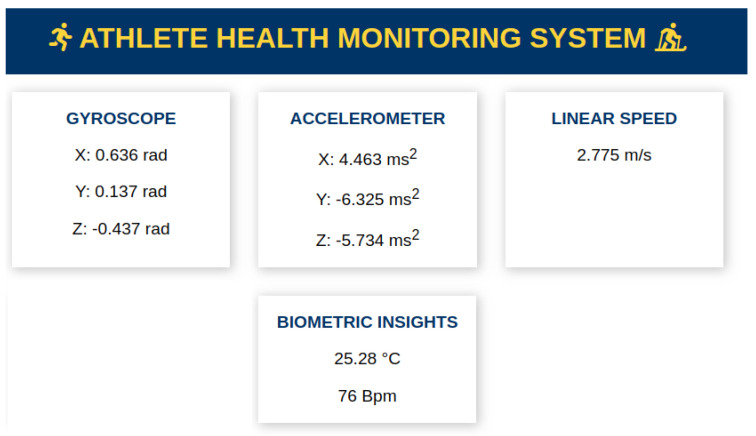
Web interface for real-time data visualization.

The web interface and backend API constitute integral components of our system, designed to enhance the user experience and ensure robust data management. The custom web interface as shown in Figure 4, meticulously crafted with HTML, CSS, and JavaScript, serves as an intuitive and user-friendly platform tailored for both athletes and health professionals. This interface provides a comprehensive visualization of the meticulously collected data, offering insightful representations and user-friendly features.

Complementing the frontend, the backend API, developed with the Flask framework in Python, serves as the backbone of our system. This API facilitates seamless and secure communication with the Firebase RTDB, establishing a dynamic connection that ensures swift and efficient data retrieval. The utilization of the Flask framework enables us to create a scalable and responsive API that aligns seamlessly with the dynamic requirements of our system.

By leveraging the capabilities of the Firebase RTDB, our backend ensures real-time updates on the web interface, empowering users with timely and accurate information. This synchronization between the web interface and backend not only enhances the overall user experience but also ensures that athletes and health professionals have access to the latest insights derived from the ongoing monitoring process.

In summary, the synergy between the custom web interface and the robust backend API is pivotal in delivering a cohesive and effective solution. The user-centric design of the web interface, coupled with the efficiency of the backend communication through Flask and Firebase, forms the technological backbone of our system, fostering an environment where data visualization, accessibility, and real-time updates converge to elevate the utility and impact of our health monitoring solution.

## 4. Alert Mechanism and Experimental Study

The alert mechanism stands as a cornerstone in our system, playing a pivotal role in ensuring athlete safety and well-being. Going beyond mere monitoring, our system boasts an advanced alert system tailored to swiftly respond to specific physiological thresholds. One such critical threshold centers around the athlete’s heart rate. For instance, during high-speed running at 100 cm/s, if the heart rate surpasses a predefined level, such as 150 BPM, an immediate alert is triggered and each triggered alert takes 2 s to reset. The system is built around predefined threshold values, such as the heart rate surpassing 150 BPM. These thresholds can be customized or adapt based on an athlete’s individual performance data, including baseline heart rate during training or running speed. The training data used to set these thresholds are derived from experiments with professional athletes or established physiological norms, ensuring precise and personalized monitoring.

To validate the effectiveness of our devices, we conducted tests utilizing a 2.7 mm mini vibration motor. Positioned inside gloves and placed against the athlete’s skin, this motor can emit vibrations in almost any circumstance, including varying temperatures, thus ensuring reliable alert delivery.

**Figure 5:**
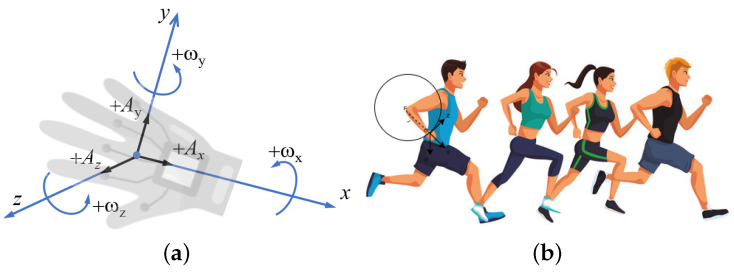
Acceleration coordinate systems used to calculate the linear speed. (**a**) Gyroscope rotation. (**b**) Athlete movement illustration.

As shown in Figure 5, when running, an athlete’s hands perform a part of a circular up-and-down movement. The elbow is the most appropriate point to take as the origin since it fluctuates around the center of the virtually formed circle. Since the origin of the circle (which in our case is the athlete’s elbow) is not fixed, there is a slight variation in the radius values, which we neglect for now.

The linear speed, a crucial metric for assessing an athlete’s velocity during physical activities, is calculated using the angular velocity data obtained from the MPU6050. To calculate the linear speed, we first determine the angular speed in rad/s on each axis x,y,z. The relationship between angular velocity (ω) and linear velocity (v) is given by the cross-product:

v=ω×r

where v is the linear velocity, ω is the angular velocity vector, and r is the position vector from the axis of rotation to the point of interest.

For a point in 3D space with coordinates $\left(r_{x},r_{y},r_{z}\right)$, if $\omega =(\omega_{x},\omega_{y},\omega_{z})$ and $r=(r_{x},r_{y},r_{z})$, the linear velocity components $v_{x}$, $v_{y}$, and $v_{z}$ can be calculated as follows:

$$v_{x}=\omega_{y}\times r_{z}-\omega_{z}\times r_{y}$$

$$v_{y}=\omega_{z}\times r_{x}-\omega_{x}\times r_{z}$$

$$v_{z}=\omega_{x}\times r_{y}-\omega_{y}\times r_{x}$$

This results in a linear velocity vector $v=(v_{x},v_{y},v_{z})$. Finally, the linear speed is given by the magnitude of the linear velocity vector:

$$v=\sqrt{v_{x}^{2}+v_{y}^{2}+v_{z}^{2}}$$

Due to the inherent noise in sensor data, we applied two smoothing techniques to improve data quality: a moving average filter with a window size of 5 and a Gaussian filter with a sigma value of 2. The moving average filter was chosen for its simplicity and effectiveness in reducing random fluctuations by averaging data points within a defined window. The window size of 5 was selected to balance between smoothing the data and preserving enough detail to accurately reflect the underlying trends. The Gaussian filter was used to provide a more refined smoothing effect by applying a weighted average based on a Gaussian distribution, which helps to better handle noise while maintaining signal integrity. The sigma value of 2 was chosen to control the extent of smoothing, offering a compromise between over-smoothing and insufficient noise reduction. We present the results of each method in separate tables and graphs to clearly compare their effects on the data.

The moving average and Gaussian filter both serve as effective smoothing techniques, but they have distinct advantages and limitations. While the moving average filtering algorithm is straightforward to implement and computationally inexpensive, making it ideal for datasets with stable noise patterns and for identifying overall trends without being affected by short-term fluctuations, the Gaussian filtering algorithm offers more effective noise reduction, particularly for Gaussian noise, by assigning higher weights to values near the center of the window, and this results in better preservation of important signal features, such as edges and peaks, and a smoother transition with less distortion compared to the moving average.

In Table 1, which displays the athlete’s physiological and environmental data during the resting phase, we can clearly conclude that all data are relatively stable. The heart rate is around 75 BPM, SpO2 around 95%, body temperature around 35 °C, and speed around 0.5 cm/s. The alert value is zero everywhere, which is normal.

Table 2 shows the data variation when the athlete starts walking. These data show that the heart rate exhibits small variations. The SpO2 level remains around 95%, similar to the resting phase. The body temperature, captured from the sensor, is around 34 °C, and the linear speed reaches a maximum value of 62.94 cm/s, which is within normal limits. The alert value is still zero since the threshold has not been reached yet.

In the last phase (running), illustrated in Table 3, we observe a significant variation in the heart rate value, which reaches a maximum of 158 BPM. The speed also increases rapidly to around 292 cm/s (2.92 m/s). The SpO2 value remains constant around 96% during this phase. The most important observation is the change in the alert value, which occasionally triggers during running to signal the athlete to slow down or take a rest. This table illustrates the alert system, which was activated when the athlete exceeded the predefined thresholds for heart rate and speed, indicating that our system is functioning as intended.

We chose to present speed in cm/s instead of m/s to highlight the presence of gyroscope drift, even when there is no actual movement. Converting the speed into m/s would misleadingly suggest a perfect zero value, which does not accurately represent the minor fluctuations detected by the gyroscope.

In Table 4, we apply a moving average filter with a window size of 5 to the raw data during the resting phase. The resulting data exhibit more consistency and less deviation. Specifically, the SpO2 values are consistently 97, the heart rate remains around 77%, and the body temperature is approximately 35 °C with only minor decimal variations. The maximum speed value, initially around 1 cm/s, is smoothed to below 0.5 cm/s. This filtering method effectively reduces noise, providing a clearer representation of the athlete’s performance metrics.

Table 5 presents the filtered data using the same moving average method during the walking phase. As observed, the deviations between each reading are significantly reduced. This indicates that the filter performs well on less noisy data.

The final analysis in Table 6 shows the smoothed data during the running phase. The consistency and reduced deviation between readings demonstrate the efficacy of this filter. The SpO2 value is approximately 96%, the maximum heart rate is now 150, and the peak speed is smoothed to 198 cm/s compared to 292 cm/s in the raw data.

**Figure 6:**
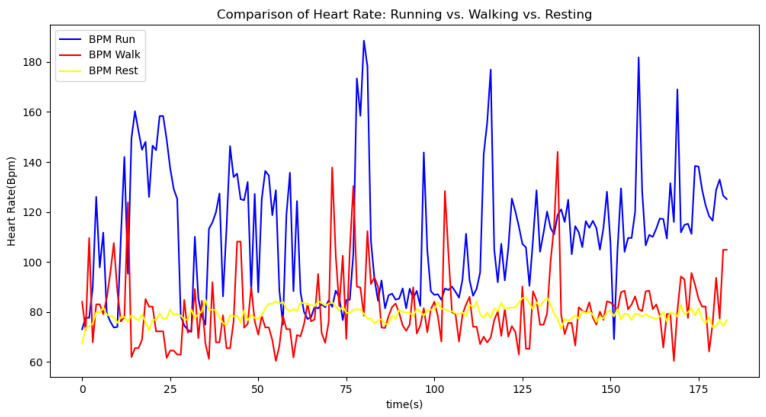
Heart rate values during different phases.

In summary, the moving average filtering method consistently reduces noise and provides a clearer, more accurate representation of physiological metrics across different phases of activity. In Table 7, Table 8 and Table 9, the raw data are filtered using a Gaussian filter for the resting, walking, and running phases, respectively. Compared to the tables related to moving average filtering, we observe that the noise is significantly reduced, and the instantaneous deviations approach zero. This indicates that the Gaussian filter performs better than the moving average method. This improvement is also visually evident in Figure 6.

**Figure 7:**
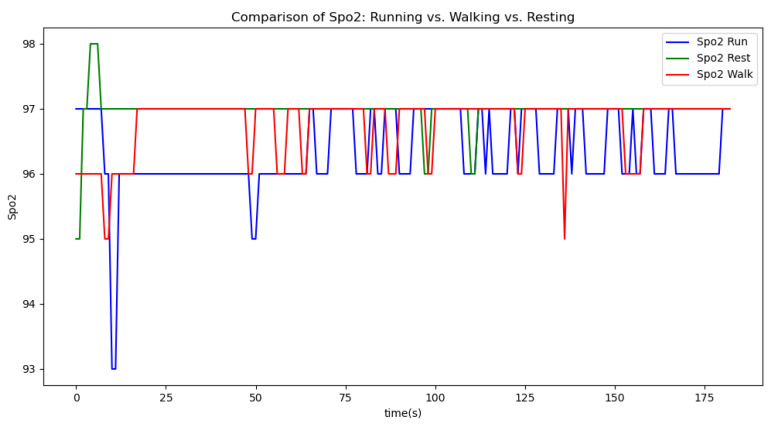
SpO2 values during different phases.

**Figure 8:**
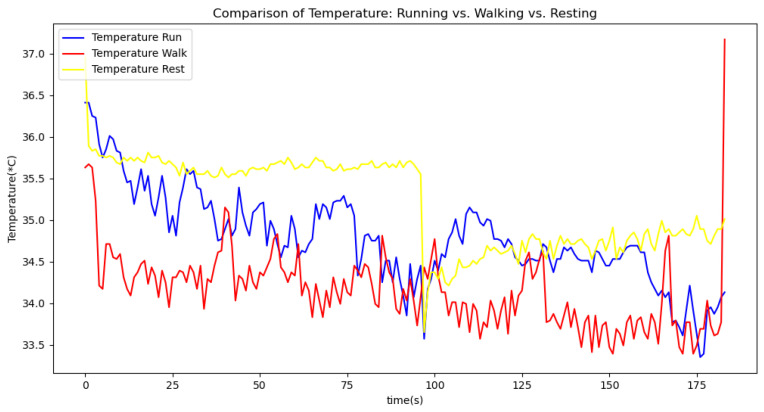
Body temperature values during different phases.

**Figure 9:**
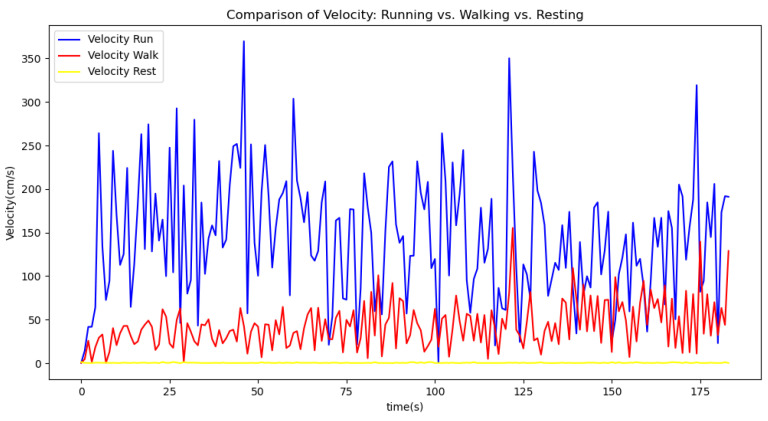
Speed values during different phases.

**Figure 10:**
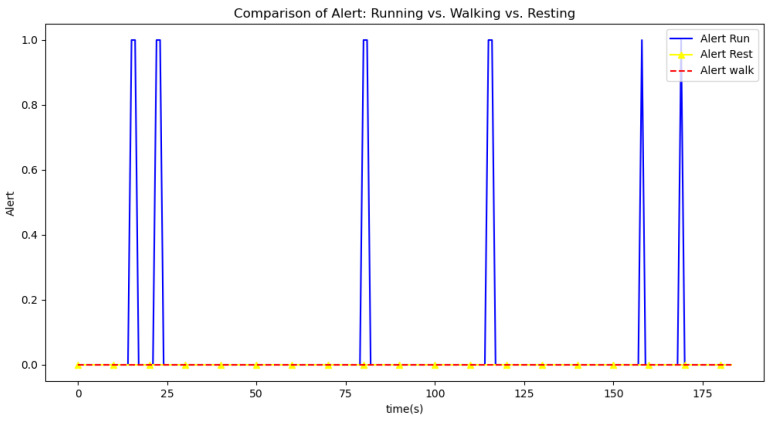
Alert signal during different phases.

Figure 6, Figure 7, Figure 8, Figure 9 and Figure 10 display comparisons of the raw data charts for heart rate, SpO2, body temperature, and speed alerts, respectively, across different phases of activity. These figures provide a visual representation of the data variations during each activity phase. Samples of 30 data points have been used to create tables summarizing key statistics and trends for each physiological parameter; these can be found in Table 1, Table 2 and Table 3.

**Figure 11:**
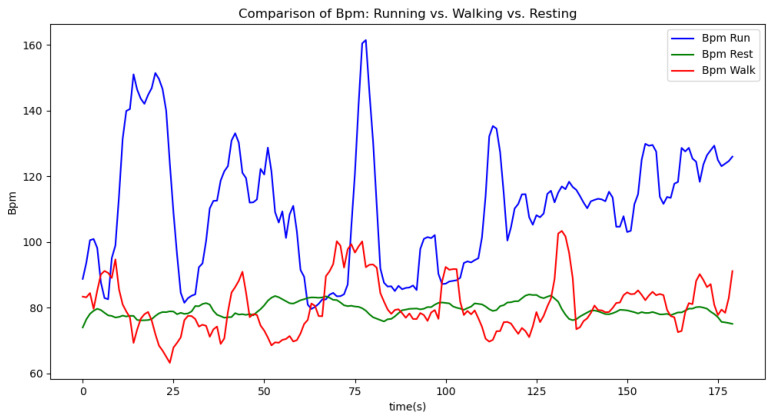
Heart rate values during different phases (moving average).

**Figure 12:**
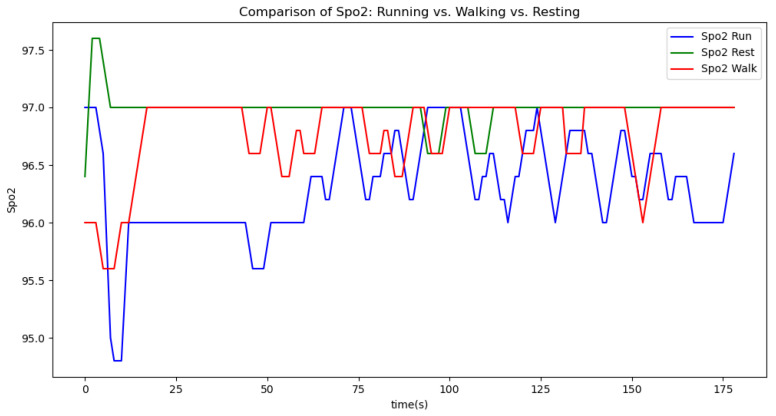
SpO2 values during different phases (moving average).

**Figure 13:**
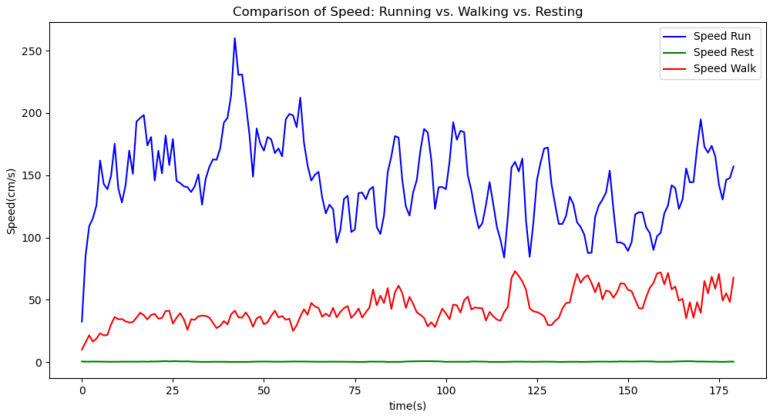
Speed values during different phases (moving average).

Figure 11, Figure 12 and Figure 13 compare the moving average smoothed data charts across different phases of activity. These visualizations help us eliminate some of the sudden spikes observed during testing. As shown in Figure 13, the speed of the athletes during the resting phase appears as an almost perfectly flat line along the x-axis, indicating the ideal speed for this phase.

**Figure 14:**
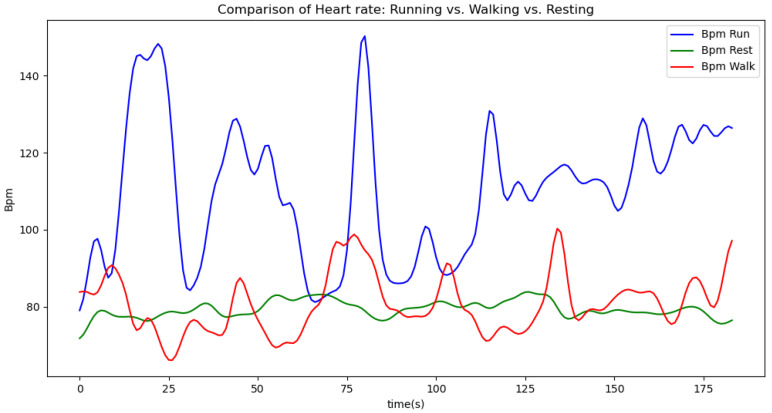
Heart rate values during different phases (Gaussian filter).

**Figure 15:**
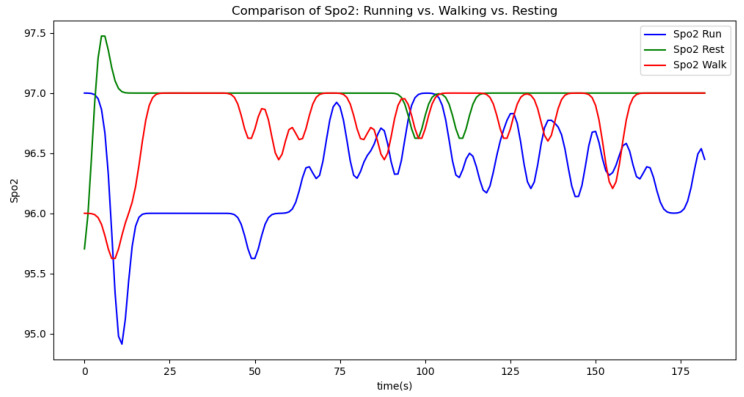
SpO2 values during different phases (Gaussian filter).

**Figure 16:**
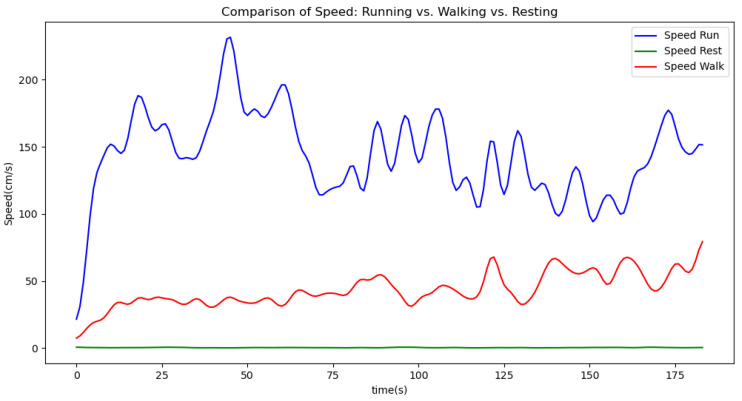
Speed values during different phases (Gaussian filter).

In the Gaussian filter data charts, Figure 14, Figure 15 and Figure 16 present a comparison of the Gaussian filter-smoothed data across different phases of activity. As illustrated, the Gaussian filter outperforms the moving average method in reducing noise and delivering a clearer, smoother representation of the data. This superior performance is evident in the sharper transitions and better preservation of significant signal features, making the Gaussian filter a more effective tool for analyzing physiological data.

After comparing the filtered data for each physiological parameter across different phases, we proceed to analyze the raw data alongside outputs from both the moving average and Gaussian filter methods for each parameter during every phase.

**Figure 17:**
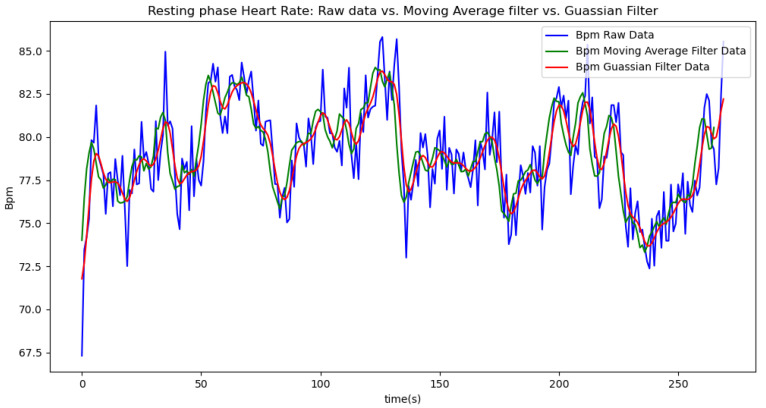
Heart rate values during the resting phase.

Figure 17 illustrates the comparison of raw heart rate data with data filtered using both the moving average and Gaussian filter methods. It is evident that both filters effectively suppress sudden spikes in the data. However, the Gaussian filter demonstrates superior performance by providing a smoother and more accurate representation of the heart rate data.

**Figure 18:**
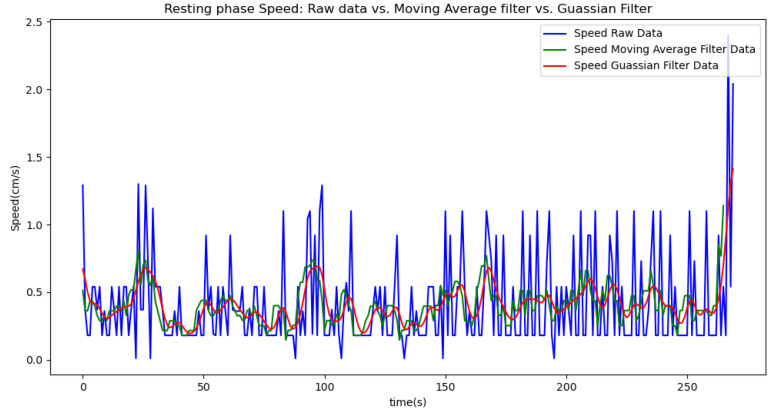
Speed values during the resting phase.

**Figure 19:**
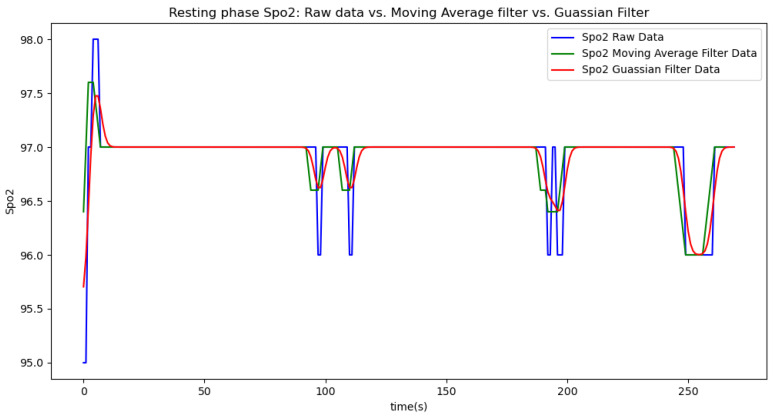
SpO2 values during the resting phase.

**Figure 20:**
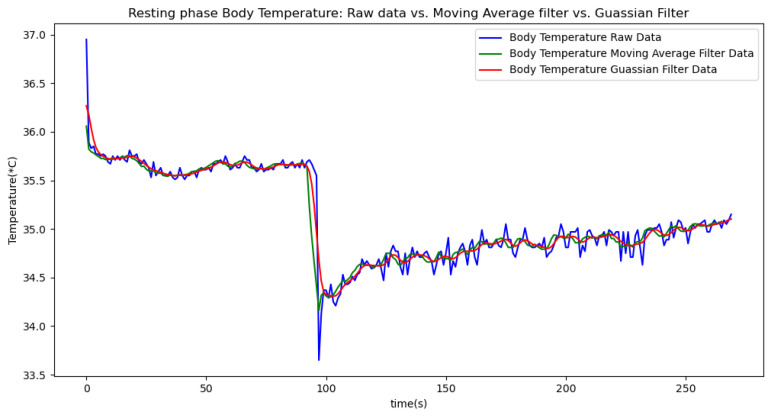
Body temperature values during the resting phase.

Figure 18, Figure 19 and Figure 20 depict similar comparisons for speed, SpO2, and body temperature, respectively. Each plot presents the original raw data alongside the filtered outputs. These comparisons consistently highlight the Gaussian filter’s capability to effectively reduce noise while preserving essential features of the physiological data.

Figure 21, Figure 22, Figure 23, Figure 24, Figure 25, Figure 26, Figure 27 and Figure 28 specifically illustrate these comparisons during both the walking and running phases, highlighting the effectiveness of the Gaussian filter across different activities and phases of the study.

**Figure 21:**
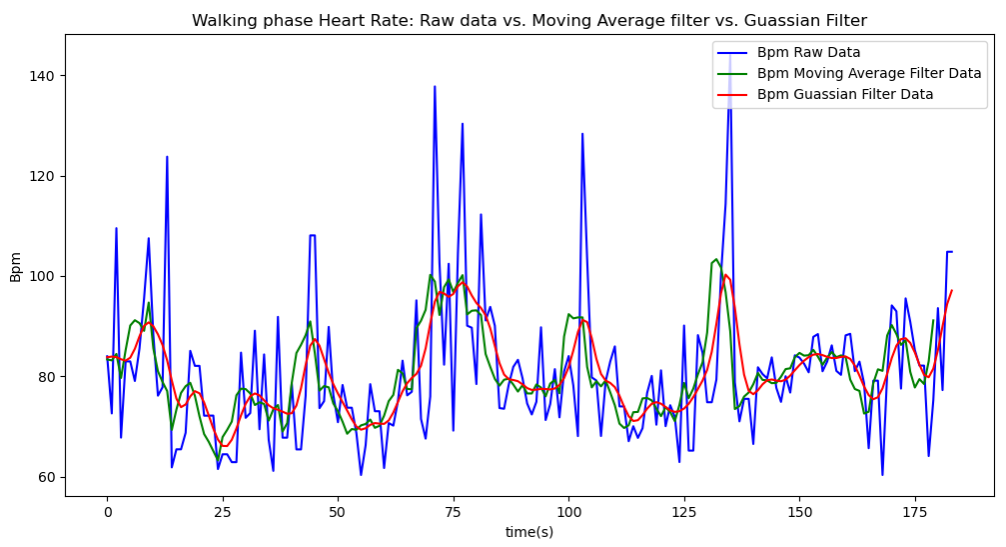
Heart rate values during the walking phase.

In Figure 21, which shows the raw values alongside the filtered ones, it is evident that sudden spikes are effectively mitigated by both filters. For instance, the spike recorded around the 134th second of our testing indicated a heart rate of approximately 145 BPM, which is unusually high for someone walking. The moving average filter reduced this value to around 110 BPM, while the Gaussian filter brought it down further to 100 BPM, both of which are closer to the recently recorded normal value of approximately 95 BPM. By significantly reducing these spikes, the filters help prevent false alerts during the athlete’s exercise.

**Figure 25:**
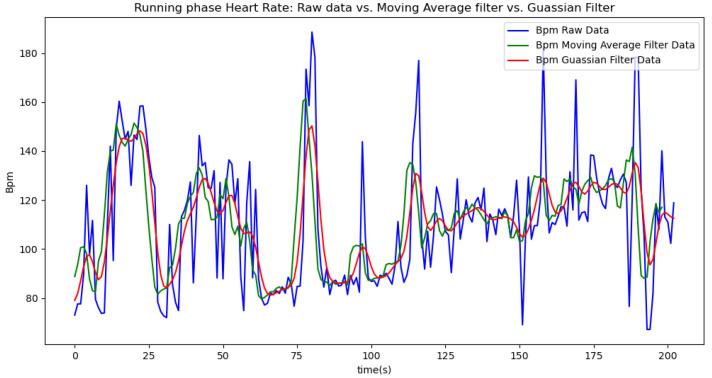
Heart rate values during the running phase.

Another example can be observed in Figure 25, during the running phase. Here, the application of the moving average filter resulted in only three alert, compared to the raw data, which triggered seven alerts. This implies that four out of the seven alerts were false when using the raw data. The Gaussian filter further improved the situation by validating only one out of the seven alerts, demonstrating superior performance in eliminating false positives.

Overall, these figures underscore the importance of using advanced filtering techniques, such as the Gaussian filter, to enhance the accuracy and reliability of health monitoring systems during various physical activities. The significant reduction in false alerts ensures that the system provides more trustworthy data, which is crucial for the safety and performance monitoring of athletes.

Our alert mechanism transcends conventional methods, fostering a proactive and responsive approach to athlete safety. By providing immediate, tangible cues, our system empowers athletes to adjust their pace wisely, thereby reducing potential health risks associated with strenuous activities. This real-time alert feature underscores our commitment to enhancing athlete well-being and implementing proactive safeguards in sports and physical training.

Analyzing various graphs reveals a clear proportional relationship between velocity and heart rate across different activity phases. Although body temperature did not exhibit a distinct pattern, its variability suggests an increase during high-intensity sports activities. Meanwhile, SpO2 levels remained constant throughout all phases, indicating limited utility in predicting certain aspects of athlete performance.

### 4.1. Challenges

During the experimentation, several challenges were encountered. Firstly, the movement of sensors such as the MLX9064 and MAX30100 during running led to issues with sensor stability, resulting in occasional false peaks in the recorded data. These fluctuations affected the accuracy of our measurements during dynamic activities.

Additionally, the experimental setup was tested on a single individual, which limits the generalizability of our findings to diverse physiological profiles. Future studies should consider testing across a broader sample size to account for variability in athlete responses and to ensure the robustness and reliability of the developed device.

Achieving accurate speed calculations presented another challenge. Relying solely on the MPU6050 sensor proved insufficient. Integrating a GPS module could provide more precise and reliable speed measurements, particularly during high-speed activities, where precision is crucial. This combination would likely improve the overall accuracy of the data.

Another significant issue was related to I2C communication. Initially, we observed random data from the MLX9064 sensor when it was coupled with other sensors operating on the I2C protocol. After extensive investigation, we identified the problem: the MAX30100 operates at 4 kHz while the MLX9064 operates at 1 kHz. To resolve this, we modified the I2C bus speed of the MAX30100 to 1 kHz in the original C++ libraries, which resulted in stable and accurate data readings from both sensors. Another limitation of our system is its high degree of domain specificity, as it is primarily designed for runner athletes. For example, swimmers may encounter issues with sensor stability underwater, which could affect the accuracy of data collection. Additionally, swimmers exhibit different arm movements compared to runners, which could negatively impact the precision of gyroscope data and motion-based speed calculations.

Overall, addressing these challenges will enhance the performance and applicability of the device, making it a more reliable tool for monitoring athletic performance across different activities and individual profiles. Future efforts should focus on refining sensor stability, expanding the sample size for testing, and integrating additional technologies such as GPS to improve the accuracy and reliability of measurements.

### 4.2. Future Extensions

The inherent adaptability and versatility of this system open up exciting possibilities for future extensions that could significantly amplify its impact. One promising avenue is the development of personalized exercise plans for athletes, leveraging the wealth of data collected through the system’s comprehensive monitoring. By incorporating advanced analytics and machine learning algorithms, the system could evolve into a dynamic platform capable of providing individualized exercise recommendations. These recommendations would be finely tuned to an athlete’s specific health parameters, historical performance data, and real-time physiological feedback, thereby optimizing training regimens tailored to each athlete’s unique needs and goals with precision.

Moreover, the integration of predictive modeling could enhance the system’s functionality by forecasting potential areas for improvement or vulnerability to injuries. This proactive approach would allow athletes and their trainers to implement preventive measures, promoting long-term health and sustained peak performance.

Additionally, expanding the system to include collaborative features could facilitate communication and data sharing among athletes, coaches, and healthcare professionals. This collaborative aspect would foster a holistic approach to athlete well-being, enabling a seamless exchange of insights and recommendations for further fine-tuning training plans. Furthermore, future studies will explore the integration of artificial intelligence and machine learning techniques, focusing on training and adapting the alert system for various athlete profiles. Exploring the integration of emerging technologies, such as augmented reality (AR) or virtual reality (VR), could further enhance the overall user experience. Immersive training environments and interactive feedback mechanisms could be incorporated, creating engaging and effective training sessions for athletes.

The system’s foundation lays the groundwork for numerous future enhancements. By embracing personalization, advanced analytics, collaborative features, and emerging technologies, the system could evolve into a sophisticated and holistic platform that not only optimizes athletic performance but also contributes to the overall well-being and resilience of athletes in their pursuit of excellence.

## 5. Conclusions

This work introduces a sophisticated IoMT-based health monitoring and alert system meticulously crafted for athletes, showcasing the seamless integration of cutting-edge technologies. The combination of the ESP8266, Firebase, MAX30100, MPU6050, and MLX9064 sensors, along with a bespoke web interface and a Flask-based backend API, forms a robust ecosystem that facilitates real-time collection, storage, and visualization of health data. The immediate alert mechanism and incorporation of linear speed calculation enhance the safety and comprehensive monitoring of athletes during physical activities. Based on our findings, no clear correlation was observed between heart rate and SpO2 levels during the experiments. The system effectively adhered to the “220-age” formula, with athletes’ heart rates staying within safe limits throughout the test sessions. Moreover, the Gaussian filter demonstrated superior performance compared to the moving average filter in handling time-series data, producing smoother and more accurate results. Notably, without any filtering, only approximately 65% of alarms were true positives. In contrast, with the Gaussian filter applied, the system achieved a 100% true positive rate, indicating no false negatives. It is important to note that while the gyroscope data from the MPU6050 sensor may exhibit instability, this issue is scheduled for resolution in future iterations. In this paper, we applied moving average and Gaussian filters to stabilize the data, thereby reducing false alerts (false positive predictions), both of which demonstrated promising results. This commitment to refining data accuracy underscores our dedication to continuous improvement in athlete health monitoring within the dynamic realms of sports and fitness. Additionally, the integration of a well-defined training protocol enables tailored adjustments to training intensity and duration, optimizing performance and minimizing the risk of overexertion and injury.

## Figures and Tables

**Figure 22:**
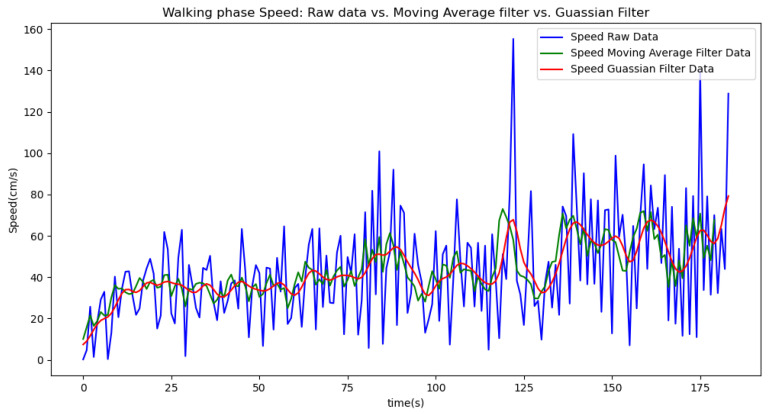
Speed values during the walking phase.

**Figure 23:**
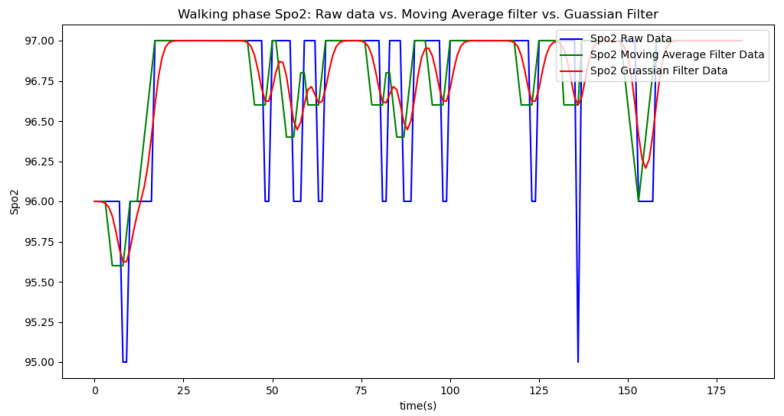
SpO2 values during the walking phase.

**Figure 24:**
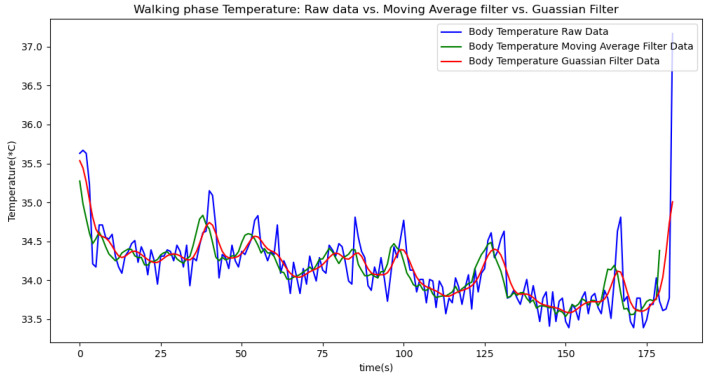
Body temperature values during the walking phase.

**Figure 26:**
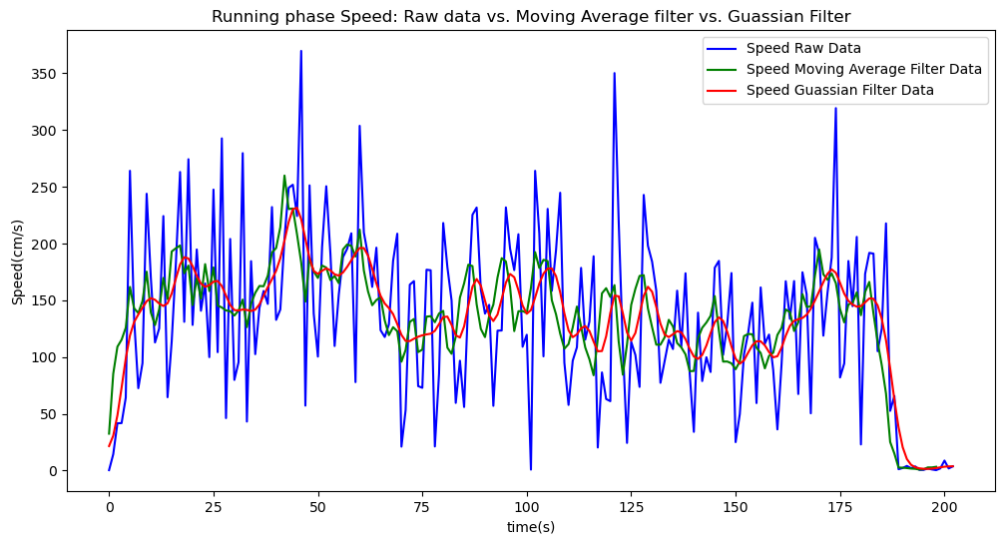
Speed values during the running phase.

**Figure 27:**
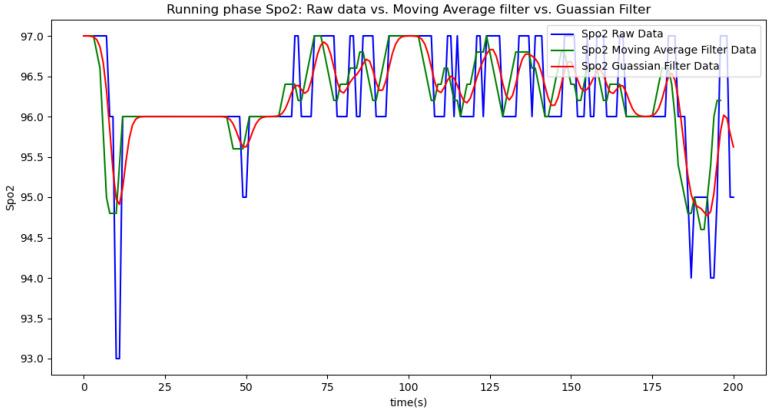
SpO2 values during the running phase.

**Figure 28:**
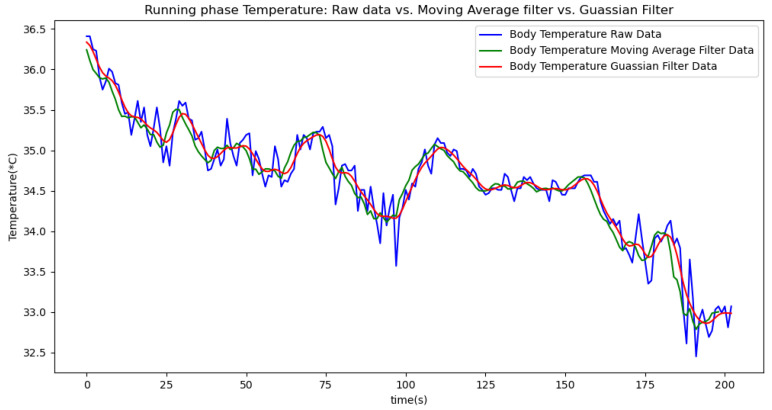
Body temperature values during the running phase.

**Table 1:** The thirty raw data points of the athletes without any physical activity (resting).

Index	Heart Rate (BPM)	SpO2	Ambient Temp (°C)	Body Temp (°C)	Speed (cm/s)	Alert
1	67.31	95.0	32.09	36.95	1.29	0
2	73.46	95.0	32.23	35.89	0.37	0
3	74.21	97.0	32.23	35.83	0.18	0
4	75.25	97.0	32.25	35.85	0.18	0
5	79.82	98.0	32.25	35.77	0.54	0
6	79.67	98.0	32.31	35.77	0.54	0
7	81.83	98.0	32.27	35.75	0.37	0
8	78.82	97.0	32.31	35.77	0.54	0
9	78.34	97.0	32.35	35.75	0.18	0
10	77.85	97.0	32.35	35.69	0.36	0
11	75.54	97.0	32.39	35.67	0.18	0
12	77.87	97.0	32.39	35.75	0.18	0
13	77.96	97.0	32.39	35.71	0.54	0
14	75.98	97.0	32.39	35.75	0.37	0
15	78.72	97.0	32.41	35.71	0.18	0
16	77.51	97.0	32.41	35.75	0.54	0
17	76.62	97.0	32.43	35.71	0.18	0
18	78.91	97.0	32.41	35.69	0.54	0
19	75.88	97.0	32.47	35.81	0.54	0
20	72.51	97.0	32.47	35.75	0.18	0
21	76.94	97.0	32.45	35.75	0.37	0
22	76.73	97.0	32.53	35.77	0.54	0
23	79.28	97.0	32.49	35.69	0.01	0
24	77.25	97.0	32.55	35.67	1.30	0
25	77.31	97.0	32.49	35.71	0.37	0
26	80.88	97.0	32.53	35.67	0.37	0
27	78.77	97.0	32.57	35.63	1.29	0
28	79.13	97.0	32.55	35.53	0.72	0
29	78.46	97.0	32.53	35.69	0.01	0
30	76.99	97.0	32.57	35.55	1.12	0

**Table 2:** The thirty raw data points of the athletes when walking.

Index	Heart Rate (BPM)	SpO2	Ambient Temp (°C)	Body Temp (°C)	Speed (cm/s)	Alert
1	84.0	96.0	33.39	35.63	0.23	0
2	72.61	96.0	33.43	35.67	4.70	0
3	109.53	96.0	33.39	35.63	25.73	0
4	67.79	96.0	33.39	35.23	1.41	0
5	82.98	96.0	33.35	34.21	18.33	0
6	82.98	96.0	33.31	34.17	29.18	0
7	79.07	96.0	33.27	34.71	32.96	0
8	85.55	96.0	33.25	34.71	0.37	0
9	95.56	95.0	33.21	34.55	13.06	0
10	107.52	95.0	33.19	34.53	40.27	0
11	88.26	96.0	33.19	34.59	20.66	0
12	76.15	96.0	33.09	34.31	34.42	0
13	77.84	96.0	33.11	34.17	42.66	0
14	123.78	96.0	33.07	34.09	42.87	0
15	61.86	96.0	32.99	34.31	31.23	0
16	65.46	96.0	32.97	34.37	21.79	0
17	65.46	96.0	32.89	34.47	24.88	0
18	68.76	97.0	32.87	34.51	38.45	0
19	85.09	97.0	32.85	34.23	44.25	0
20	82.09	97.0	32.83	34.43	48.92	0
21	82.09	97.0	32.79	34.33	41.70	0
22	72.16	97.0	32.79	34.07	15.17	0
23	72.18	97.0	32.77	34.39	21.44	0
24	72.18	97.0	32.73	34.25	61.90	0
25	61.52	97.0	32.67	33.95	53.51	0
26	64.47	97.0	32.67	34.31	22.53	0
27	64.47	97.0	32.61	34.31	17.63	0
28	62.9	97.0	32.61	34.39	49.50	0
29	62.9	97.0	32.57	34.37	62.94	0
30	84.73	97.0	32.57	34.25	1.82	0

**Table 3:** The thirty raw data points of the athletes when running.

Index	Heart Rate (BPM)	SpO2	Ambient Temp (°C)	Body Temp (°C)	Speed (cm/s)	Alert
1	73.04	97.0	32.63	36.41	0.36	0
2	77.6	97.0	32.67	36.41	14.36	0
3	77.6	97.0	32.69	36.25	41.65	0
4	89.65	97.0	32.67	36.23	41.80	0
5	126.0	97.0	32.67	35.91	64.25	0
6	97.8	97.0	32.65	35.75	264.14	0
7	111.68	97.0	32.65	35.85	133.73	0
8	79.42	97.0	32.67	36.01	72.56	0
9	76.08	96.0	32.65	35.97	94.56	0
10	73.7	96.0	32.63	35.83	243.92	0
11	73.93	93.0	32.61	35.81	169.97	0
12	109.79	93.0	32.59	35.59	112.85	0
13	141.98	96.0	32.57	35.45	125.20	0
14	95.27	96.0	32.57	35.47	224.25	0
15	149.34	96.0	32.53	35.19	64.61	0
16	160.32	96.0	32.49	35.39	113.97	1
17	152.36	96.0	32.53	35.61	182.78	1
18	144.88	96.0	32.47	35.35	263.09	0
19	148.05	96.0	32.45	35.53	131.04	0
20	126.0	96.0	32.43	35.19	274.35	0
21	146.5	96.0	32.41	35.05	128.31	0
22	144.71	96.0	32.39	35.27	194.79	0
23	158.34	96.0	32.37	35.53	140.80	1
24	158.42	96.0	32.37	35.27	164.90	1
25	149.04	96.0	32.33	34.85	99.93	0
26	137.48	96.0	32.33	35.05	247.58	0
27	129.2	96.0	32.27	34.81	104.30	0
28	125.22	96.0	32.25	35.21	292.72	0
29	78.3	96.0	32.27	35.39	46.21	0
30	74.52	96.0	32.25	35.61	204.08	0

**Table 4:** Smoothed data applying moving average filtering (the thirty data points of the athletes when resting).

Index	Heart Rate (BPM)	SpO2	Ambient Temp (°C)	Body Temp (°C)	Speed (cm/s)	Alert
1	76.48	97.00	32.25	35.82	0.36	0
2	78.16	97.60	32.26	35.79	0.36	0
3	79.08	97.60	32.28	35.78	0.43	0
4	79.70	97.60	32.30	35.76	0.43	0
5	79.30	97.40	32.32	35.75	0.40	0
6	78.48	97.20	32.33	35.73	0.33	0
7	77.68	97.00	32.36	35.73	0.29	0
8	77.51	97.00	32.37	35.71	0.29	0
9	77.04	97.00	32.38	35.71	0.33	0
10	77.21	97.00	32.39	35.72	0.29	0
11	77.61	97.00	32.40	35.73	0.36	0
12	77.36	97.00	32.41	35.73	0.36	0
13	77.55	97.00	32.41	35.72	0.36	0
14	77.53	97.00	32.43	35.73	0.40	0
15	76.29	97.00	32.44	35.74	0.40	0
16	76.17	97.00	32.45	35.74	0.36	0
17	76.19	97.00	32.47	35.75	0.43	0
18	76.27	97.00	32.48	35.75	0.33	0
19	76.54	97.00	32.50	35.73	0.48	0
20	77.50	97.00	32.50	35.72	0.52	0
21	78.29	97.00	32.52	35.70	0.52	0
22	78.70	97.00	32.53	35.67	0.67	0
23	78.67	97.00	32.54	35.64	0.81	0
24	78.91	97.00	32.53	35.65	0.55	0
25	78.85	97.00	32.55	35.61	0.70	0
26	78.04	97.00	32.57	35.60	0.74	0
27	78.47	97.00	32.58	35.60	0.59	0
28	78.14	97.00	32.59	35.60	0.55	0
29	78.27	97.00	32.60	35.57	0.62	0
30	78.90	97.00	32.61	35.57	0.43	0

**Table 5:** Smoothed data applying moving average filtering (the thirty data points of the athletes when walking).

Index	Heart Rate (BPM)	SpO2	Ambient Temp (°C)	Body Temp (°C)	Speed (cm/s)	Alert
1	83.18	96.00	33.37	34.98	15.87	0
2	84.47	96.00	33.34	34.79	21.52	0
3	79.67	96.00	33.31	34.61	16.45	0
4	85.23	95.80	33.28	34.47	18.78	0
5	90.14	95.60	33.25	34.53	23.17	0
6	91.19	95.60	33.22	34.62	21.46	0
7	90.61	95.60	33.19	34.54	21.76	0
8	89.07	95.60	33.16	34.43	30.21	0
9	94.71	95.80	33.13	34.34	36.18	0
10	85.58	96.00	33.09	34.29	34.37	0
11	81.02	96.00	33.05	34.25	34.59	0
12	78.88	96.00	33.01	34.28	32.69	0
13	77.06	96.20	32.96	34.35	31.84	0
14	69.33	96.40	32.91	34.38	32.12	0
15	73.37	96.60	32.88	34.40	35.66	0
16	76.70	96.80	32.85	34.39	39.64	0
17	78.04	97.00	32.83	34.31	37.70	0
18	78.72	97.00	32.81	34.29	34.30	0
19	76.14	97.00	32.78	34.29	37.83	0
20	72.03	97.00	32.75	34.20	38.74	0
21	68.50	97.00	32.73	34.19	34.91	0
22	66.96	97.00	32.69	34.24	35.40	0
23	65.11	97.00	32.66	34.24	41.01	0
24	63.25	97.00	32.63	34.27	41.22	0
25	67.89	97.00	32.61	34.33	30.88	0
26	69.35	97.00	32.58	34.35	35.57	0
27	70.99	97.00	32.55	34.37	39.26	0
28	76.23	97.00	32.54	34.32	34.38	0
29	77.54	97.00	32.52	34.34	25.90	0
30	77.47	97.00	32.49	34.27	34.43	0

**Table 6:** Smoothed data applying moving average filtering (the thirty data points of the athletes when running).

Index	Heart Rate (BPM)	SpO2	Ambient Temp (°C)	Body Temp (°C)	Speed (cm/s)	Alert
1	93.73	97.00	32.67	36.11	85.24	0
2	100.55	97.00	32.67	36.00	109.11	0
3	100.91	97.00	32.66	35.95	115.30	0
4	98.20	96.80	32.66	35.90	125.85	0
5	87.74	96.60	32.65	35.88	161.78	0
6	82.96	95.80	32.64	35.89	142.95	0
7	82.58	95.00	32.63	35.84	138.77	0
8	95.10	94.80	32.61	35.73	149.30	0
9	98.93	94.80	32.59	35.63	175.24	0
10	114.06	94.80	32.57	35.50	139.38	0
11	131.34	95.40	32.55	35.42	128.18	0
12	139.85	96.00	32.54	35.42	142.16	0
13	140.43	96.00	32.52	35.40	169.74	0
14	150.99	96.00	32.49	35.41	151.10	0
15	146.32	96.00	32.47	35.41	193.05	1
16	143.56	96.00	32.46	35.35	195.91	1
17	142.03	96.00	32.43	35.28	198.32	0
18	144.72	96.00	32.41	35.31	173.86	0
19	146.79	96.00	32.39	35.26	180.63	0
20	151.40	96.00	32.37	35.19	145.75	0
21	149.60	96.00	32.36	35.19	169.60	0
22	146.50	96.00	32.33	35.10	151.50	1
23	139.87	96.00	32.31	35.04	181.89	1
24	123.85	96.00	32.29	35.06	158.15	0
25	108.94	96.00	32.27	35.21	178.98	0
26	96.00	96.00	32.25	35.31	145.44	0
27	84.56	96.00	32.25	35.47	143.66	0
28	81.52	96.00	32.24	35.51	141.06	0
29	82.92	96.00	32.22	35.50	140.44	0
30	83.66	96.00	32.21	35.41	136.53	0

**Table 7:** Smoothed data applying Gaussian filter (the thirty data points of the athletes when resting).

Index	Heart Rate (BPM)	SpO2	Ambient Temp (°C)	Body Temp (°C)	Speed (cm/s)	Alert
1	72.70	95.98	32.20	36.17	0.60	0
2	74.25	96.44	32.22	36.04	0.50	0
3	76.00	96.92	32.24	35.92	0.44	0
4	77.55	97.29	32.26	35.84	0.42	0
5	78.60	97.47	32.28	35.79	0.41	0
6	79.04	97.47	32.30	35.77	0.41	0
7	78.91	97.36	32.32	35.75	0.38	0
8	78.45	97.21	32.34	35.74	0.34	0
9	77.93	97.10	32.35	35.72	0.31	0
10	77.55	97.04	32.37	35.72	0.30	0
11	77.38	97.01	32.38	35.72	0.31	0
12	77.35	97.00	32.39	35.72	0.33	0
13	77.39	97.00	32.40	35.73	0.34	0
14	77.41	97.00	32.41	35.73	0.36	0
15	77.33	97.00	32.42	35.73	0.37	0
16	77.10	97.00	32.43	35.73	0.38	0
17	76.74	97.00	32.44	35.74	0.39	0
18	76.39	97.00	32.45	35.74	0.39	0
19	76.26	97.00	32.47	35.75	0.40	0
20	76.48	97.00	32.48	35.74	0.41	0
21	76.95	97.00	32.49	35.73	0.45	0
22	77.50	97.00	32.51	35.71	0.51	0
23	78.00	97.00	32.52	35.69	0.58	0
24	78.40	97.00	32.52	35.68	0.63	0
25	78.66	97.00	32.53	35.66	0.67	0
26	78.73	97.00	32.54	35.64	0.68	0
27	78.61	97.00	32.55	35.62	0.67	0
28	78.42	97.00	32.56	35.61	0.65	0
29	78.32	97.00	32.58	35.60	0.62	0
30	78.41	97.00	32.59	35.59	0.58	0

**Table 8:** Smoothed data applying Gaussian filter (the thirty data points of the athletes when walking).

Index	Heart Rate (BPM)	SpO2	Ambient Temp (°C)	Body Temp (°C)	Speed (cm/s)	Alert
1	83.94	96.00	33.39	35.44	9.05	0
2	83.84	96.00	33.38	35.26	11.66	0
3	83.41	95.99	33.36	35.03	14.62	0
4	83.13	95.96	33.34	34.81	17.27	0
5	83.72	95.91	33.31	34.66	19.08	0
6	85.50	95.81	33.28	34.58	19.98	0
7	87.98	95.70	33.25	34.56	20.79	0
8	90.07	95.62	33.22	34.54	22.54	0
9	90.76	95.62	33.19	34.50	25.53	0
10	89.98	95.70	33.16	34.44	29.11	0
11	88.37	95.82	33.12	34.37	32.23	0
12	86.23	95.92	33.09	34.31	33.99	0
13	83.17	96.00	33.05	34.29	34.08	0
14	79.19	96.09	33.00	34.31	33.17	0
15	75.56	96.22	32.96	34.34	32.63	0
16	73.86	96.40	32.92	34.37	33.50	0
17	74.40	96.60	32.88	34.37	35.50	0
18	76.01	96.78	32.85	34.36	37.24	0
19	77.05	96.90	32.83	34.33	37.55	0
20	76.62	96.96	32.80	34.30	36.71	0
21	74.80	96.99	32.78	34.27	36.10	0
22	72.22	97.00	32.75	34.24	36.64	0
23	69.58	97.00	32.72	34.23	37.67	0
24	67.40	97.00	32.69	34.23	37.96	0
25	66.14	97.00	32.66	34.25	37.41	0
26	66.11	97.00	32.63	34.28	36.86	0
27	67.37	97.00	32.60	34.31	36.62	0
28	69.62	97.00	32.58	34.33	36.10	0
29	72.22	97.00	32.56	34.34	34.94	0
30	74.51	97.00	32.54	34.33	33.55	0

**Table 9:** Smoothed data applying Gaussian filter (the thirty data points of the athletes when running).

Index	Heart Rate (BPM)	SpO2	Ambient Temp (°C)	Body Temp (°C)	Speed (cm/s)	Alert
1	83.94	96.00	33.39	35.44	9.05	0
2	83.84	96.00	33.38	35.26	11.66	0
3	83.41	95.99	33.36	35.03	14.62	0
4	83.13	95.96	33.34	34.81	17.27	0
5	83.72	95.91	33.31	34.66	19.08	0
6	85.50	95.81	33.28	34.58	19.98	0
7	87.98	95.70	33.25	34.56	20.79	0
8	90.07	95.62	33.22	34.54	22.54	0
9	90.76	95.62	33.19	34.50	25.53	0
10	89.98	95.70	33.16	34.44	29.11	0
11	88.37	95.82	33.12	34.37	32.23	0
12	86.23	95.92	33.09	34.31	33.99	0
13	83.17	96.00	33.05	34.29	34.08	0
14	79.19	96.09	33.00	34.31	33.17	0
15	75.56	96.22	32.96	34.34	32.63	0
16	73.86	96.40	32.92	34.37	33.50	0
17	74.40	96.60	32.88	34.37	35.50	0
18	76.01	96.78	32.85	34.36	37.24	0
19	77.05	96.90	32.83	34.33	37.55	0
20	76.62	96.96	32.80	34.30	36.71	0
21	74.80	96.99	32.78	34.27	36.10	0
22	72.22	97.00	32.75	34.24	36.64	0
23	69.58	97.00	32.72	34.23	37.67	0
24	67.40	97.00	32.69	34.23	37.96	0
25	66.14	97.00	32.66	34.25	37.41	0
26	66.11	97.00	32.63	34.28	36.86	0
27	67.37	97.00	32.60	34.31	36.62	0
28	69.62	97.00	32.58	34.33	36.10	0
29	72.22	97.00	32.56	34.34	34.94	0
30	74.51	97.00	32.54	34.33	33.55	0

## Data Availability

The original contributions presented in the study are included in the paper, further inquiries can be directed to the corresponding author.
